# Effect of adenosine and adenosine receptor antagonist on Müller cell potassium channel in Rat chronic ocular hypertension models

**DOI:** 10.1038/srep11294

**Published:** 2015-06-11

**Authors:** Zijian Yang, Ping Huang, Xiaohong Liu, Shouyue Huang, Lianfu Deng, Zhe Jin, Shuo Xu, Xi Shen, Xunda Luo, Yisheng Zhong

**Affiliations:** 1 Department of Ophthalmology, Ruijin Hospital Affiliated Medical School, Shanghai Jiaotong University, 197 Ruijin Er Road, 200025, Shanghai, China; 2 Shanghai Institute of Traumatology and Orthopaedics, 197 Ruijin Er Road, 200025, Shanghai, China; 3 Department of Ophthalmology, Scheie Eye Institute, Perelman School of Medicine, University of Pennsylvania, Philadelphia, PA 19104, USA; 4 Department of Pathology and Laboratory Medicine, Temple University Hospital, Philadelphia, PA 19140, USA

## Abstract

Müller cells are principal glial cells in rat retina and have attracted much attention in glaucoma studies. However, it is not clear whether adenosine and adenosine receptor (AR) antagonists play any roles in the regulation of potassium channels in Müller cells and subsequently in the promotion of glutamine synthetase (GS) and L-Glutamate/L-Aspartate Transporter (GLAST) functions. We found that chronic ocular hypertension (COH) in rat down-regulated Müller cells Kir2.1, Kir4.1, TASK-1, GS and GLAST expressions and attenuated the peak of inward potassium current. Retinal ganglion cells (RGC) count was lower in the COH rats than that in the sham operation animals. Intravitreal injection of selective A_2A_ AR antagonist SCH442416 up-regulated Müller cell Kir4.1, TASK-1, GS and GLAST expressions and enhanced inward potassium currents compared with those in the COH rats with vehicle control. Meanwhile, the RGC count was higher following intravitreal injection of SCH442416 in the COH rats than that after vehicle injection. The fact that PKA inhibitor H-89 blocked these SCH442416 effects suggested that the PKA signaling pathway was involved in the observed ocular responses following the intravitreal SCH442416 injection.

Glaucoma is a leading cause of blindness in the world and the mechanisms of glaucoma still have not been fully understood. The functions of glial cells, especially those of Müller cells, have been attracting increasing attentions among glaucoma neuroprotection. Some studies have shown that the loss of appropriate interaction with the extracellular matrix might be an important signal within the retina which triggers axon degeneration and RGC apoptosis.

Müller cells, a principal type of glial cells in mammalian retinae, are specialized radial glial cells which span the entire thickness of the retina, and are related closely to structure and function of retinal blood vessels and neurons^1^. The surface-to-volume ratio of Müller cell processes is very high, and these processes can contact almost all neuronal elements. There are abundant of different ion channels on Müller cells, such as ligand receptors, transmembrane transporter molecules, and enzymes^2^. One notable character of Müller cell membrane is high voltage-gated potassium channel which mainly include inwardly rectifying channels (Kir family, mainly Kir4.1 and Kir2.1 channel) and tandem-pore channels (TASK channel)^3^, calcium and neurotransmitter activities, and high K^+^ conductance. GS has been found in Müller cells and has been used as a specific marker for these cells^4^. Müller cells, which respond to virtually all pathological alterations of the retina-that includ photic damage, retinal trauma, ischemia, retinal detachment, glaucoma, diabetic retinopathy, and age-related macular degeneration, play a key role in regulating ion and water homeostasis and synaptic activity through neurotransmitter recycling and gliotransmitter release^5^,^6^.

Adenosine can be found extensively in both intracellular and extracellular fluids. Biological effects of adenosine are mediated through adenosine receptors (ARs), which are characterized as G-protein linked receptors and can be grouped into four subtypes, i.e.-A_1_, A_2A_, A_2B_ and A_3_ receptors. It has already been confirmed biologically and pharmacologically that all four types of ARs are expressed in the retina^7^,^8^. Neurotransmitter release from synaptic terminals, including that for glutamate, is inhibited following activation of A_1_ receptors, and subsequent reduction of calcium influx in response to the action potential propagated to the terminals^9^. A_2A_ receptors are mainly expressed in the striatum, especially in GABAergic striatopallidal projection neurons and cholinergic interneurons^10^. Activation of A_2A_ receptors, however, promotes the release of neurotransmitters (including glutamate). Some studies have demonstrated that adenosine regulates potassium channel function in the kidney^11^ and A_2A_ antagonists provide neuroprotection to the cerebral cortex^12^,^13^. However, it is still not clear whether adenosine and AR antagonists can regulate potassium channels in Müller cells in the retina. The purpose of this study was to elucidate the effects of and the pathways used by adenosine and AR antagonist, especially the selective A_2A_ antagonist SCH442416, on the regulation of Müller cell potassium channel function.

## Result

### Kir2.1, Kir4.1, TASK-1, GS and GLAST expressions in rat chronic ocular hypertension (COH) models

**Figure 1:**
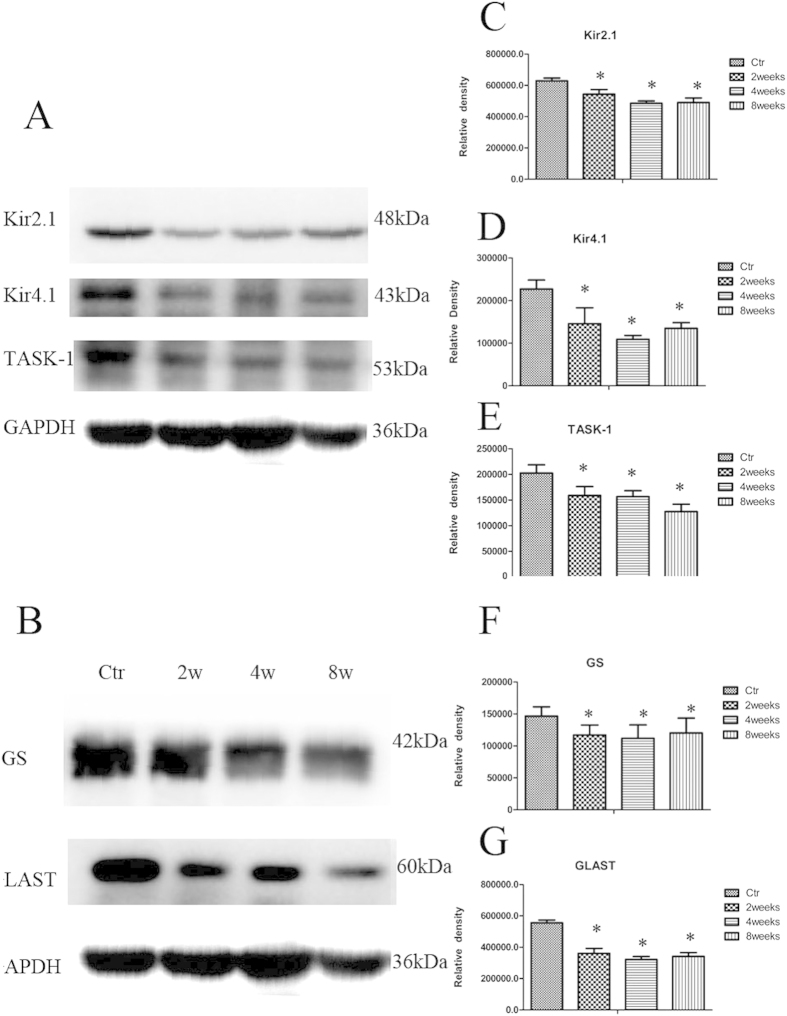
Changes in three potassium channels, GS and GLAST protein expressions in control and COH rat retina at different time points.

Kir2.1, Kir4.1, TASK-1 protein and mRNA expressions in rat retinae were evaluated by western-blot and real-time PCR. Two, four and eight weeks following the induction of COH, Kir2.1, Kir4.1 and TASK-1 protein expressions decreased significantly compared to those in rats with sham operation (Fig. 1). At second, fourth and eighth week after operation, Kir2.1 protein expressions decreased by 14.6%, 23.8% and 26.4% respectively (n = 6; *p* = 0.000, 0.000 and 0.000); Kir4.1 protein expressions decreased by 59.7%, 50.6 and 37.0% (n = 6; *p* = 0.080, 0.000 and 0.000); TASK-1 protein expressions decreased by 26.0%, 26.0% and 38.9% (n = 6; *p* = 0.000, 0.000 and 0.000), respectively (Fig. 1).

Two, four and eight weeks following COH, mRNA expressions of retina Kir2.1 were down-regulated by 22.4%, 24.2% and 26.1% (n = 6; *p* = 0.036, 0.037 and 0.007, respectively) compared to the controls with shamed-operation; Kir4.1 mRNA expressions was down-regulated by 20.7%, 39.8% and 51.1% (n = 6; *p* = 0.006, 0.005 and 0.001, respectively); TASK-1 mRNA was down-regulated by 19.9%, 18.1% and 61.2% (n = 6; *p* = 0.037, 0.038 and 0.014, respectively).

GS and GLAST protein and mRNA expressions were also evaluated by western-blot and real-time PCR. GS and GLAST protein expressions two, four and eight weeks following COH were found to be significantly decreased compared to the controls. GS protein expressions decreased by 20.0%, 23.6% and 17.9% (n = 6; *p*  = 0.014, 0.005 and 0.026, respectively) at these time points; GLAST protein expressions decreased by 35.0%, 42.1% and 38.6% (n = 6; *p* = 0.000, 0.000 and 0.000, respectively) (Fig. 1).

Two, four and eight weeks after COH, the mRNA expressions of retina GS was down-regulated by 16.0%, 10.4% and 30.0% (n = 6; *p*  = 0.040, 0.034 and 0.000, respectively); the mRNA expressions of GLAST was down-regulated by 38.2%, 51.2% and 49.7% (n = 6; *p*  = 0.005, 0.000 and 0.003, respectively).

**Figure 2:**
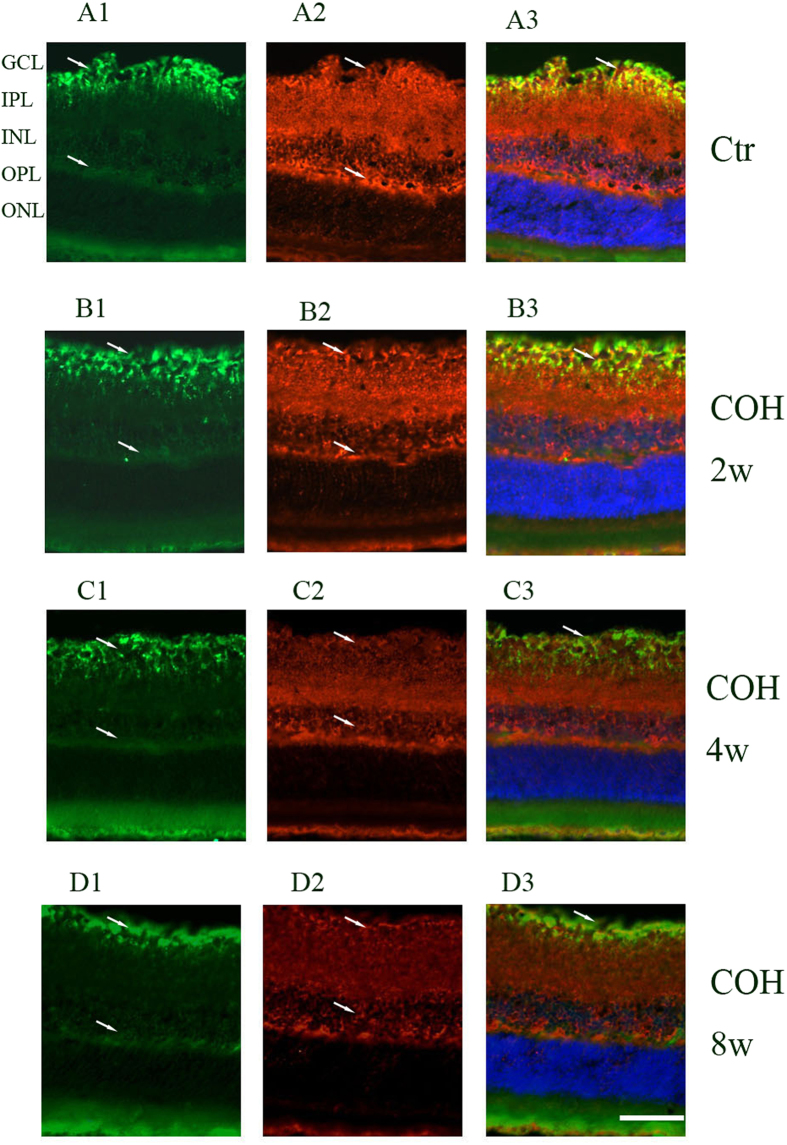
Changes of potassium channel Kir2.1 and GS protein expressions in control and COH rat retina Müller cells.

**Figure 3:**
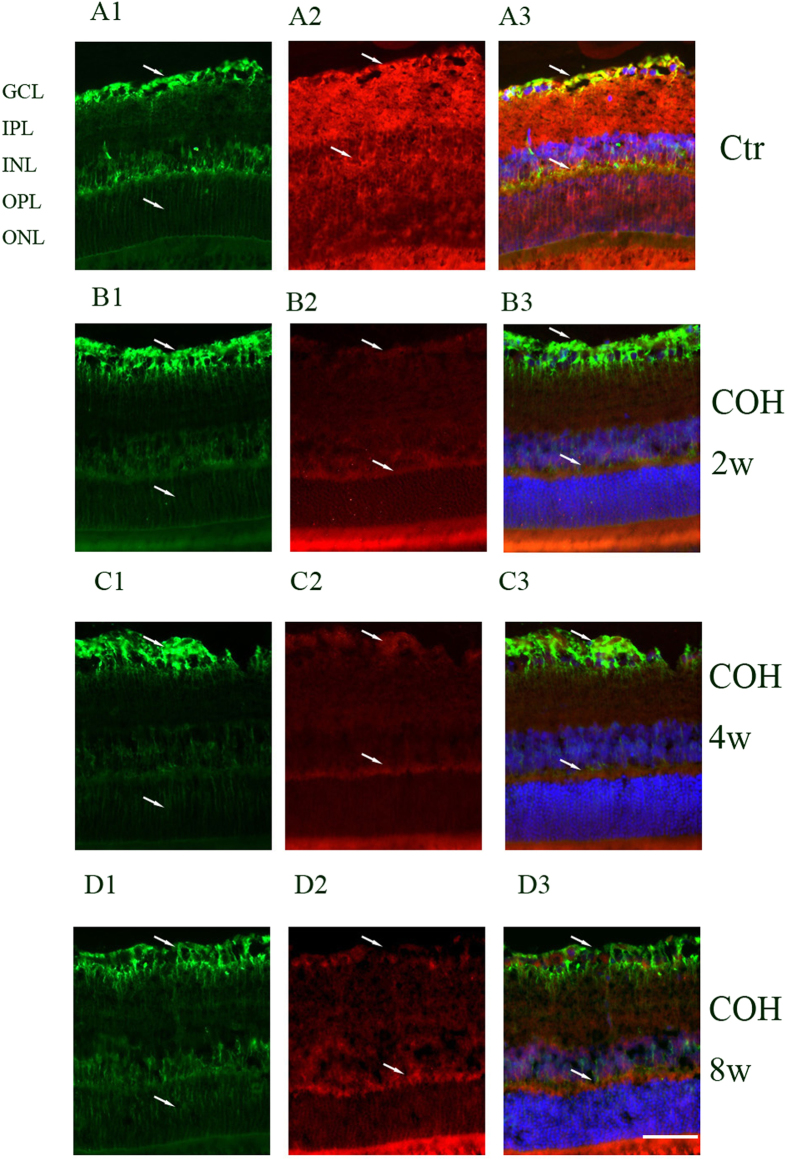
Changes of potassium channel Kir4.1 and GS protein expressions in control and COH rat retina Müller cells.

**Figure 4:**
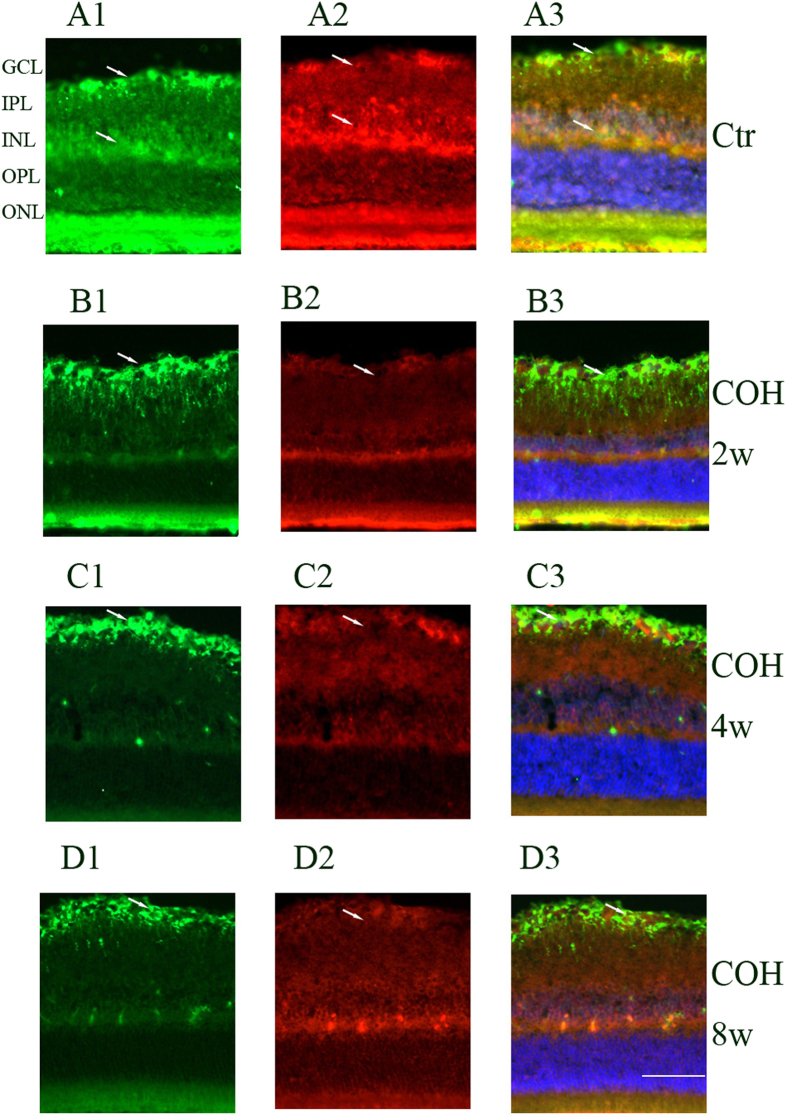
Changes of potassium channel TASk-1 and GS protein expressions in control and COH rat retina Müller cells.

Inwardly rectifying channels of the Kir family mainly include Kir2.1 and Kir4.1. Kir2.1 channels were distributed rather evenly in the membrane between endfoot and soma; Kir4.1 channels were mainly located at the vitread endfoot, the outer plexiform layer (OPL), the perivascular membrane areas and the microvilli. Tandem-pore (TASK-1) channels showed a subcellular distribution similar to that of the Kir2.1 channels in addition to a prominent expression in the microvilli. GS was identified in Müller cells, whereas GLAST was identified throughout the retina. We double stained retinal slices anti-Kir2.1, anti-Kir4.1, anti-TASK-1 and anti-GS to reveal the relationship between GS and the potassium channels protein expressions. As shown in Figs 2, 3, 4, 2 weeks, 4 weeks and 8 weeks following the COH surgeries, Müller cells Kir2.1, Kir4.1, TASK-1 and GS protein expressions decreased significantly compared with that in control.

### Changes of Kir2.1, Kir4.1, TASK-1, GS and GLAST expressions by adenosine and adenosine receptor antagonists in the rat COH model

**Figure 5:**
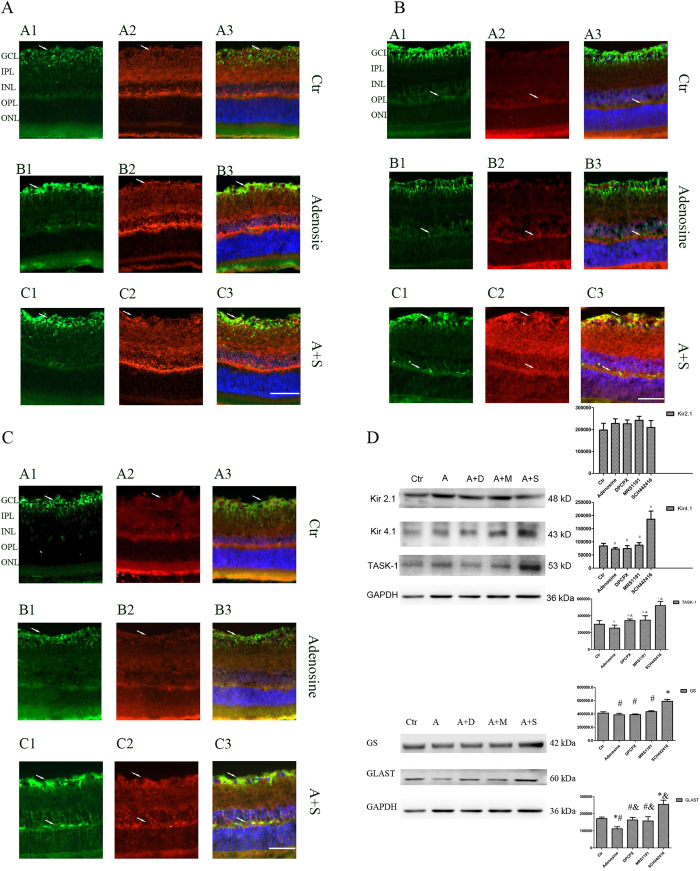
Effect of adenosine and adenosine receptor antagonist on Kir2.1, Kir4.1, TASK-1, GS and GLAST protein expressions in COH rat retina.

Adenosine, adenosine + DPCPX, adenosine + MRS1191, adenosine + SCH442416 and vehicle (5% DMSO) were intravitreally injected into the right eyes of the COH rats. These animals were sacrificed after two weeks and the retinae dissected and used for experiment. It was shown by western-blot that Kir2.1, Kir4.1 and TASK-1 protein expressions had no significant change in adenosine, adenosine + DPCPX and adenosine + MRS1191 groups compared with the vehicle control group (n = 6; *p* = 0.243, 0.249, 0.121; *p* =  0.194, 0.536, 0.979; *p* = 0.680, 0.810, 0.520, respectively) (Fig. 5), which suggests that adenosine, DPCPX and MRS1191 had no effect on Kir2.1, Kir4.1 and TASK-1 protein expressions in the retina of the COH rats. Kir2.1 protein expressions was mildly increased by 0.1% in adenosine + SCH442416 group compared with vehicle control group (n = 6; *p*  = 0.983), however, Kir4.1 and TASK-1 protein expressions were increased significantly by 120.9% and 79.3% respectively in adenosine + SCH442416 group compared with the vehicle control group (n = 6; *p* = 0.010 and 0.000, respectively) (Fig. 5).

GS and GLAST protein expressions were decreased by 6.3% and 34.7% in the adenosine group, respectively (n = 6; *p* = 0.030 and 0.000, respectively). No significant changes were observed in the adenosine + DPCPX and adenosine + MRS1191 groups. However, in the adenosine + SCH442416 group, GS and GLAST protein expressions increased by 48.3% and 42.8%, respectively (n = 6; *p* = 0.000 and 0.000, respectively) (Fig. 5).

Immunohistochemical experiments showed Kir2.1 protein expressions among the groups were not significantly different. Compared with the vehicle control group and the adenosine group, Kir4.1, TASK-1, GS and GLAST protein expressions increased in the adenosine + SCH442416 group. Kir4.1 protein expressions were markedly increased at the endfood and OPL; TASK-1 protein expressions increased significantly in general; meanwhile, GS protein expressions increased at the endfoot (Fig. 5).

### Counting of RGC

**Figure 6:**
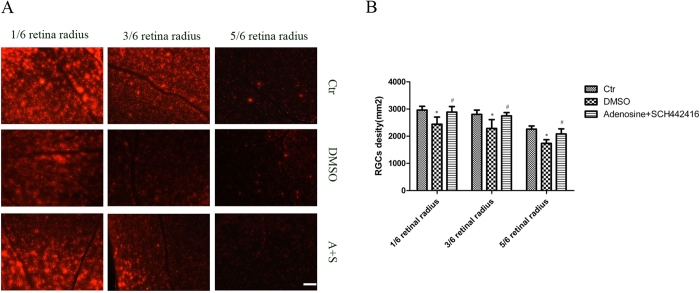
Changes of RGCs density induced by COH and SCH442416.

Two weeks after adenosine+SCH442416 intravitreal injection (One week after bilateral SC retrograde DiI label), RGC counting was performed. In the control eyes (sham-operated rat), a higher RGC density was observed in the retinal near the optic nerve and it declined from the central to the peripheral retina (2963 ± 137/mm^2^ in 1/6 retinal radius, 2805 ± 154/mm^2^ in 3/6 retinal radius and 2261 ± 117/mm^2^ in 5/6 retinal radius, respectively). The number of RGC in the vehicle injection COH rat retina (2442 ± 262/mm^2^ in 1/6 retinal radius, 2289 ± 321/mm^2^ in 3/6 retinal radius and 1735 ± 136/mm^2^ in 5/6 retinal radius, respectively) was markedly decrease compared with the control (n = 6; *p* = 0.001, 0.022 and 0.000, respectively). RGCs were better preserved following intravitreal injection of SCH442416 in the COH rats (2887 ± 202/mm^2^ in 1/6 retinal radius, 2748 ± 124/mm^2^ in 3/6 retinal radius and 2082 ± 187/mm^2^ in 5/6 retinal radius, respectively) than that following the vehicle injection (n = 6; *p* = 0.002, 0.036, 0.001, respectively) (Fig.6).

### PKA signaling pathway mediated the SCH442416 effect

**Figure 7:**
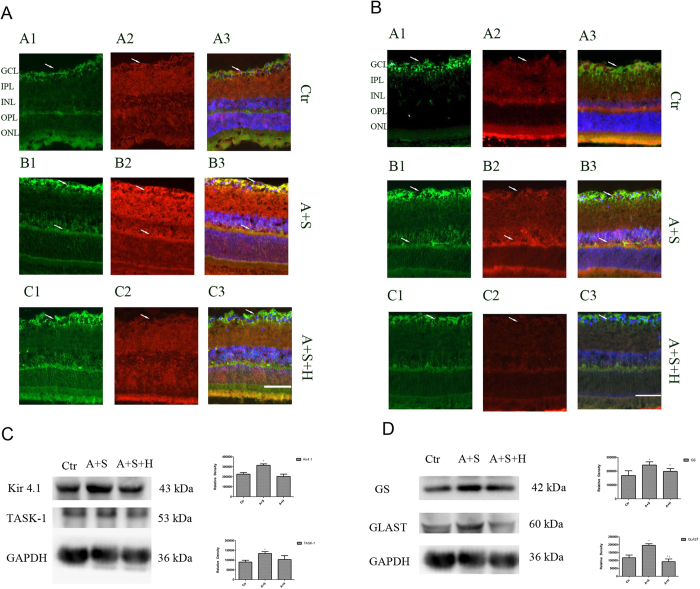
PKA signaling pathway mediates the SCH442416 induced increase of Kir4.1, TASK-1, GS and GLAST protein expressions in COH rat retina.

Selective adenosine A_2A_ receptors, couple to pathognostic G-protein and regulate cyclic adenosine monophosphate (cAMP) level through the adenylate cyclase. Blockage of adenosine A_2A_ receptor may activate adenylate cyclase and subsequently protein kinase A (PKA)^14^. The signaling pathway may be responsible for the increase in Kir4.1 and TASK-1 protein expressions. In order to explore the signaling pathway of the selective A_2A_ antagonist SCH442416 on Kir4.1 and TASK-1 expressions, we used PKA inhibitor H-89 (10μM) to block PKA. Adenosine+SCH442416+H-89 were intravitreally injected into right eyes of COH rat. Rats were sacrificed, retinae dissected and used for experiments two weeks later. The results demonstrated that the PKA inhibitor H-89 influenced the SCH442416-induced increase of Kir4.1 and TASK-1 protein expressions in the retina of COH rats. Administration of SCH442416 induce Kir4.1 and TASK-1 protein expressions markedly increased, however, administration of SCH442416 + H-89 induced Kir4.1 and TASK-1 protein expressions no significant change compared with the retina of COH rats with the vehicle injection (n = 6; *p* = 0.578, *p* = 0.386). We also explore the relationship of the changes between GS, GLAST and potassium channel. The study showed GS and GLAST display a similar change to Kir4.1 and TASK-1. Administration of SCH442416 induce GS and GLAST protein expressions markedly increased, however, administration of SCH442416+ H-89 induced GS and GLAST protein expressions no significant change (n = 6; *p* = 0.680, *p* = 0.110) (Fig. 7).

Immunofluorescence labeling showing the Kir4.1 and TASK-1 protein expressions in retinal slices taken from the vehicle control, SCH442416 or SCH442416+H-89 injected rat eyes. Coexpression of TASK-1 and GS protein yield a yellow merge signal. Administration of SCH442416, Kir4.1, TASK-1 and GS markedly increased fluorescence compared with vehicle control, however, administration of H-89+SCH442416, Kir4.1, TASK-1 and GS significantly decreased fluorescence compared with administration of SCH442416 (Fig. 7).

### Suppression of potassium currents in Müller cells of the COH rats

**Figure 8:**
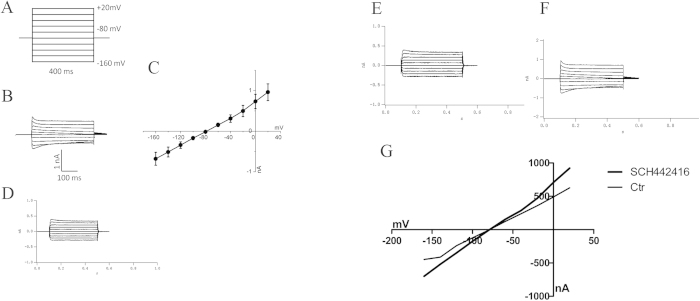
Suppression of potassium currents in Müller cell of the COH rats and Müller cell Kir currents changes induced by SCH442416.

Kir currents were recorded in isolated retinal Müller cells following COH-inducing and sham surgeries. The Müller cells potassium currents induced by a series of hyperpolarized voltage pulses from a holding potential of −160 mV in an increment of 20 mV are shown in Fig. 8. We found a down-regulation of potassium currents in Müller cells of the COH rats. Müller cells of the COH rats strongly down-regulated transmembrane potassium currents. The peak current amplitudes were decreased to approximately 45% of control level (45.4  ± 9.8%) (n = 6; *p* = 0.005) (Fig. 8).

### Effect of SCH442416 on Müller cell Kir currents

Previous biochemical and electrophysiological studies have suggested that A_2A_ AR antagonists may regulate the function of potassium channels in rat kidneys^11^. We therefore explored whether adenosine and adenosine receptor antagonist affected retinal Müller cells potassium function. Two weeks following intravitreal SCH442416 injection, Kir currents were recorded in isolated retinal Müller cells of the rat COH model. The results showed SCH442416 (100nM) significantly increased the peak potassium currents in isolated Müller cells from the COH rat retinae by 55.8  ± 8.5% compared with those recorded from the isolated Müller cells treated with the vehicle injection (n = 6; *p* = 0.014) (Fig. 8).

## Discussion

Müller cells are a major type of glial cells in the retina. Double labeling of GS and potassium channel proteins provided us with a method to observe changes in Müller cells Kir2.1, Kir4.1 and TASK-1 protein expressions in the COH rat. In response to COH, as we observed in the present study, Müller cell potassium channels protein expressions, especially those for Kir4.1 and TASK-1, were dramatically down-regulated. The decreased expressions of Kir4.1 and TASK-1 proteins were accompanied by a decrease of Kir4.1 and TASk-1 mRNA level, suggesting that the down-regulation of Kir4.1 and TASK-1 were mainly caused by decreased transcription. Correspondingly, the Kir currents evoked by voltage steps/ramps recorded from isolated Müller cells decreased significantly as well. The study also showed that the *I*–*V* relationships of Müller cells Kir currents exhibited a weakly inward rectifying feature, which suggested that the current was mainly mediated by Kir4.1 and TASK-1 channels. These results demonstrated that COH mainly affected expressions and functions of Kir4.1 and TASK-1 channels, and less on those of Kir2.1 channels.

Müller cells can regulate synaptic transmission by releasing glutamate and adenosine^15^. The Müller cells release of adenosine is mediated by calcium-independent facilitated transport method^16^,^17^. However, Müller cells may lose these functions and promote neuronal degeneration in pathognomonic conditions^18^,^19^. Müller cell membrane is expected to be in depolarization condition when the Kir2.1, Kir4.1, TASK-1, GS and GLAST expressions are induced and potassium inward current is changed in rat COH model. In the conditions of depolarization, Müller cells release glutamate and the increased non-vesicular release of glutamate leads to excitotoxic damage to neurons^20^. Adenosine is an endogenous bioactive components and regulates physiological functions via membrane receptors. Four subtypes of ARs have been identified: A_1_, A_2A_, A_2B_ and A_3_^21^. Physiological level of adenosine is expected to be 20–200 nM. A_2B_ AR can be activated by a high-concentration of adenosine; A_2A_ AR, in contrast, can be activated by a relatively lower adenosine concentration^22^, hence a 10 μM adenosine was selected in the study^11^.

In physiological conditions, glutamate is the major excitatory neurotransmitter in mammalian central nervous system (including the retina). GS and GLAST are two primary glial enzymes functioning in the clearance of extracellular glutamate in physiological condition. These functions could deteriorate significantly in pathological condition. With the down-regulation of GS and GLAST, the neurotransmitter concentrations in the retina, including that of glutamate, are increased. Some studies suggest that it is the down-regulation of potassium channels on cell membrane that affects the efficiency of glutamate transfer functions of GS and GLAST^23^,^24^. The present study shows that COH down-regulates the expressions of Müller cells GS and GLAST proteins. Administration of SCH442416 helps to better preserve GS and GLAST expressions. These results indicate that there is an association between GS, GLAST and potassium channel expressions.

Although GS and GLAST depression were changed intertwined in some degree^25^, our study also demonstrated that GLAST expressions dropped more than that of GS in the COH rats, suggesting that GLAST may have played a more major role in glutamate level changes.

The present study demonstrated that selective A_2A_ AR antagonist SCH442416 enhenced Kir4.1 and TASK-1 protein expressions in the COH rats. However, Kir2.1 protein expressions were not affected by SCH442416. These results are consistent with findings from a previous study that different mechanisms are involved in Kir2.1 and Kir4.1 regulation^26^. Meanwhile, selective A_1_ AR antagonist DPCPX and selective A_3_ AR antagonist MRS1191 had no effect on Müller cells Kir2.1, Kir4.1 and TASK-1 protein expressions. It has been postulated that PKA is the pathway used by selective A_2A_ AR antagonist to regulate potassium channel activity^27^. Data from the present study support this hypothesis. H-89, an inhibitor of PKA, blocked the effects of selective adenosine A_2A_ receptor antagonist SCH442416 on Kir4.1 and TASK-1 protein expressions in the COH rats. Following Kir4.1 and TASk-1 expressions decreased, GS and GLAST expressions decreased simultaneously. The result showed that Müller cells potassium channel expressions covaried with GS and GLAST expressions.

As it had been shown previously, Müller cells potassium channel expressions was inhibited in the COH. Following treatment of SCH442416, Kir4.1 and TASK-1 protein expressions recovered, what changes of potassium channel function would be? As shown in the study, Müller cells Kir currents was significantly increased by SCH442416 (100 nM) in the COH rats. That means SCH442416 not only improved potassium protein expressions, but also improved potassium channels function in Müller cell.

## Materials and Method

All experimental protocols were approved by Shanghai Institute of Traumatology and Orthopaedics.

### Animals and rat glaucoma model

All experimental procedures described here were in accordance with the National Institutes of Health (NIH) guidelines for the Care and Use of Laboratory Animals. Male Sprague Dawley rats, weighing 200–250 g, were purchased from Shanghai Slack Laboratory Animals Ltd, and housed in an air-conditioned room at approximately 23 °C and 60% humidity on a 12-hr light/dark cycle. All animals were handled in accordance with the ARVO resolution for the use of animals in ophthalmic and vision research. Anaesthesia was performed with an intraperitoneal injection of xylazine and ketamine hydrochloride (10 mg/kg and 25 mg/kg, respectively). A drop of proxymetacaine hydrochloride (0.5%) was used for local anesthesia.

The COH was induced in the right eyes of the rats, however, the left eyes were not considered as control eyes. The model was reproduced following a procedure described previously^28^,^29^,^30^. The IOP was elevated by cauterizing three episcleral veins in each eye. The episcleral veins were separated and cauterized carefully in order not to damage the tissue nearby. Sham-operations, which were similar to the COH-inducing operations except for the absence of episcleral vein cauterization, were performed on the right eyes of rats free from any ocular operations or intravitreal injections.

### Drugs and Intravitreal injection

The rat pupil was dilated with tropicamide(0.5%), and 10 μM adenosine, 10 μM (8-cyclopentyl-1,3-dipropylxanthine(DPCPX)+10 μM adenosine, 100 nM 5-amino-2-(2-furyl)-7-[3-(4-methoxyphenyl)propyl]-7H-pyrazolo[4,3-e][1,2,4]triazolo[1,5-c]pyrimidine (SCH442416) +10 μM adenosine, 10 μM (3-ethyl 5-benzyl 2-methyl-6-phenyl-4-phenyl-ethynyl-1, 4-(+)-dihydropyridine-3,5-dicarboxylate, MRS 1191) +10 μM adenosine, and 10 μM H-89+100 nM SCH442416+ 10 μM adenosine were used. The drugs were first dissolved in DMSO and then diluted by ddH_2_O to 2 μl, within which the final concentration of DMSO was 5%. A Hamilton microinjector was inserted 2 mm behind the temporal limbus and directed toward the optic nerve, and the solution was injected into the vitreous space under the direct visualization through a stereoscopic microscope. Eyes that received only an injection of vehicle solution (5% DMSO) in the same manner served as vehicle controls.

### Real-time PCR

The PCR solution contained 2 μl of cDNA, specific primer set (1 μM each) and 11.5 μl of QuantiTect SYBR Green PCR Kit (Qiagen, Hilden, Germany) in a final volume of 20 μl. The following primer pairs were used: Kir2.1, sense 5’-ctctcctggctgttctttgg-3’, anti-sense 5’-atcgggcactcgtctgtaac- 3’ (product size 188 bp, Invitrogen); Kir4.1, sense 5’-caaagaagagggctgagacg-3’, anti-sense 5’-ttgagccgaatatcctcacc-3’, (product size 181 bp, Invitrogen); TASK-1 sense 5’ atggtgctcatcggtttcgt 3’, anti-sense 5’ cgtactgaggctgcgtttgc 3’ (product size 187 bp, Invitrogen); GS sense 5’ccgctcttcgtctcgttc3’, anti-sense 5’ctgcttgatgcctttgtt3’ (product size 2764 bp, Invitrogen); GLAST sense 5’ cctatgtggcagtcgttt3’, anti-sense 5’ctgtgatgggctggctaa3’ ,(product size 149 bp, Invitrogen); β-actin, sense 5’-gcgctcgtcgtcgacaacgg-3’, anti-sense 5’-gtgtggtgccaaatcttctcc-3’ (product size 248 bp, Invitrogen). The PCR parameters were initial denaturation, one cycle at 94 °C for 5 min; amplification and quantification, 40 cycles at 94 °C for 30 s, 55 °C for 30 s, and 72 °C for 30 s; melting curve, 55 °C with the temperature gradually increased up to 95°C. The mRNA expressions was normalized to the levels of β-actin mRNA as described previously^31^.

### Western-blot analysis

Nucl-Cyto-Mem Preparation Kit was used in protein extraction. Rat retinae were put on ice box, ground 20 times with 500 μl Cytosol Extraction Reagent in glass homogenizer, ice-bath 10 min, and then ground 7 more times. Homogenates were centrifuged at 800 × g for 5 min at 4 °C. Add 1/10 volume Membrane Extraction Reagment to liquid supernatant and ice bath 5 min, centrifuge at 14000 × g for 30 min at 4 °C. Deposit was lysed in 100 μl Suspension buffer. All the gels have been run under the same experimental conditions. Equal amount of protein samples with 10% SDS-PAGE Electrophoresis in a Mini-Protean 3 electrophoresis system (Bio-Rad) and electrotransferred to polyvinylidene fluoride membranes using a Mini Trans-Blot electrophoretic transfer system (Bio-Rad) for 90 min. After blocked with 5% skimmed milk at room temperature for 2 h, the membranes incubated with rabbit monoclonal anti –Kir2.1 (1:1000 dilution; Abcam), goat polyclonal antibody anti- Kir4.1 (1:300 dilution; Abcam), rabbit polyclonal antibody anti-K_2p_3.1(TASK-1, 1:200 dilution; Alomone Labs), rabbit polyclonal antibody anti-GS(1:10000 dilution; Abcam), rabbit polyclonal antibody anti-GLAST (1:200 dilution; Sigma) or mouse monoclonal antibody anti-GAPDH (1:10000 dilution, Kangchen) overnight at 4 °C, then washed with TBST for 3 × 10 min and incubated with HRP-conjugated goat anti-rabbit (1:2000; Abcam), anti-mouse IgG (1:2000; Abcam) or rabbit anti-goat (1:3000; Weiao) for 1 h at room temperature and visualized with enhanced chemofluorescence reagent (Beyotime). Pictures were taken by ImageQuant Las 4000 mini and the protein bands were quantitatively analyzed with Imageproplus (IPP) image analysis software.

### Immunohistochemistry

Rats were anesthetized and perfused with 200 ml NS and 200 ml 4% paraformaldehyde (PFA, in 0.1 M PB, pH 7.4) solution. The right eyeballs were enucleated and fixed in 4% PFA solution for 4 h, then dehydration with grade sucrose solution at 4 °C. The retinae were vertically sectioned at 10 μm thickness. Retina slices were mounted on gelatin-coated slides. The slices were blocked in 4% goat, 0.25% serum bovine serum albumin (BSA), 0.2% Triton X-100 in PBS at room temperature for 2 h, and then incubated with mouse monoclonal anti-GS (1:200 dilution; Abcam), goat polyclonal anti-GLAST (1:200 dilution; Santa cruz), rabbit monoclonal anti –Kir2.1 (1:200 dilution; Abcam), rabbit polyclonal anti-Kir4.1 (1:200 dilution; Abcam) and rabbit polyclonal anti-K_2p_3.1(TASK-1)(1:200 dilution; Alomone Labs) primary antibodies at 4 °C for 48 h. Immunoreactive proteins were visualized by incubating with FITC conjugated donkey anti-mouse IgG (1:100 dilution; Jackson Immuno-Research Laboratories), FITC conjugated donkey anti-goat (1:50; Santa Cruz) and cy3-conjugated donkey anti-rabbit IgG (1:100 dilution; Biolegend). The samples were mounted with mounting medium with DAPI (Vector Laboratories) and the immunofluorescence images were visualized with a Zeiss Imager.M1 laser-scanning microscope using a 20 × objective lens.

### Bilateral superior colliculus (SC) retrograde DiI label and quantification of RGC

Seven days after COH surgery, the DiI labeling was conducted. Bilateral SC retrograde DiI labeling was performed following the procedure described in detail previously^32^. Briefly, the DiI suspension was prepared by mixing 3 mg DiI in 1 ml saline containing 1% to 3% Triton X-100. Following the hair and skin removal, the rat cranium pierce position was located at the point behind fonticuli minor 6.4 mm, apart from the line 1.5 mm and then inserting needle 4.0 mm from the skull surface. Each point was injected 1.5 μl DiI by micro injector. Seven days after the DiI application, the rat was perfused transcardially with saline water and 4% paraformaldehyde (PFA) 30 minutes, respectively. After being postfixed in 4% PFA solution for 1 hour, the retinae were dissected and mounted on slides. Images were captured immediately by laser fluorescence microscope. RGC were counted in an area of approximately the same distance from the optic disc-1/6, 3/6 and 5/6 retinal radius. Using a digital imaging system (ImagePro 6.0).

### Müller cells dissociation

A modification of the dissociation procedures of Müller cells was used^33^. Rat retina was incubated in Hank’s solution containing papain (1.6 U/ml, Worthington Biochemical), L-cysteine (0.2 mg/ml) and bovine serum albumin (BSA, 0.2 mg/ml) for 30 min at 37 °C, the solution was bubbled continuously with O_2_ for at least 30 min and adjusted to pH 7.4 with NaOH. The retinal pieces were rinsed in Hank’s solution containing BSA and then digested in Hank’s solution containing BSA and DNAase I (200 U/ml), After rinsed with Hank’s solution three times, the retinae were mechanically dissociated with pipette, and the cell suspension was cultured into a culture dish for 2–3 hours and then mounted on an inverted microscope (IX 70; Olympus Optical).

### Whole-cell patch-clamp recordings

Whole-cell patch-clamp recordings in dissociated Müller cells from rats were conducted using a patch clamp amplifier (EPC10; HEKA Electronik, Lambrecht, Germany). Stimulation and recordings were controlled by PULSE software. Capacitive transients and series resistance errors were minimized before recording. External medium for patch clamp contained (in mM): 140 NaCl, 5.6 KCl, 1.2 MgCl2, 2.6 CaCl2, 10 HEPES, and 10 glucose (pH 7.4 with NaOH). Patch pipettes were made by pulling BF150–86-10 glass (Sutter Instrument Co.) on a P-97 flaming/brown micropipette puller (Sutter Instrument Co.) and fire polished (Model MF-830; Narishige) for recording. The pipette resistance typically had a resistance of 8–10 MΩ while filled with internal recording solution contained (in mM): NaCl 20, K-gluconate 130, CaCl2 1,MgCl2 2, EGTA 1, HEPES 10, GTP-Na 0.1, and ATP-Mg 2, adjusted topH 7.2 with KOH and to 290–300 mOsm/L. Inwardly rectifying K+-selective (Kir) currents were evoked by a series of hyperpolarized voltage pulses from a holding potential of −80 mV in increments of 20 mV, voltage steps between −160 mV and +20 mV. All experiments were carried out at room temperature (22 °C–24 °C).

### Statistics

Data were analyzed using SPSS19.0 software (SPSS, Chicago, IL, USA). The Paired-sample student t-test or one-way ANOVA were used for statistical significance. The values were presented as mean ± SD. A *p* value that was smaller than 0.05 were considered to be statistically significant.

## Additional Information

**How to cite this article**: Yang, Z. *et al.* Effect of adenosine and adenosine receptor antagonist on Müller cell potassium channel in Rat chronic ocular hypertension models. *Sci. Rep.* **5**, 11294; doi: 10.1038/srep11294 (2015).
